# Non-Coding RNAs in Normal B-Cell Development and in Mantle Cell Lymphoma: From Molecular Mechanism to Biomarker and Therapeutic Agent Potential

**DOI:** 10.3390/ijms22179490

**Published:** 2021-08-31

**Authors:** Olga Kersy, Mali Salmon-Divon, Ofer Shpilberg, Oshrat Hershkovitz-Rokah

**Affiliations:** 1 Department of Molecular Biology, Faculty of Natural Sciences, Ariel University, Ariel 40700, Israel; kersyolga@gmail.com (O.K.); malisa@ariel.ac.il (M.S.-D.); 2 Translational Research Lab, Assuta Medical Centers, Tel-Aviv 6971028, Israel; ofers@assuta.co.il; 3 Adelson School of Medicine, Ariel University, Ariel 40700, Israel; 4 Institute of Hematology, Assuta Medical Centers, Tel-Aviv 6971028, Israel

**Keywords:** microRNA, circRNA, lncRNA, B-cell lymphoma, mantle cell lymphoma

## Abstract

B-lymphocytes are essential for an efficient immune response against a variety of pathogens. A large fraction of hematologic malignancies are of B-cell origin, suggesting that the development and activation of B cells must be tightly regulated. In recent years, differentially expressed non-coding RNAs have been identified in mantle cell lymphoma (MCL) tumor samples as opposed to their naive, normal B-cell compartment. These aberrantly expressed molecules, specifically microRNAs (miRNAs), circular RNAs (circRNAs) and long non-coding RNAs (lncRNAs), have a role in cellular growth and survival pathways in various biological models. Here, we provide an overview of current knowledge on the role of non-coding RNAs and their relevant targets in B-cell development, activation and malignant transformation, summarizing the current understanding of the role of aberrant expression of non-coding RNAs in MCL pathobiology with perspectives for clinical use.

## 1. Introduction

The development of RNA sequencing (RNA-seq) technologies enabled the discovery of highly abundant and diverse classes of non-coding RNA (ncRNA) molecules [1]. These molecules lack protein-coding potential but are essential to the regulation of epigenetic, transcriptional, and post-translational mechanisms involved in homeostasis and diseases [2]. 

The species of ncRNAs mainly include small nuclear RNAs, small nucleolar RNAs, microRNAs (miRNAs), Piwi-interacting RNAs (piRNAs), long ncRNAs (lncRNAs) and circular RNAs (circRNAs) [3,4]. 

B-lymphocytes are essential for an efficient immune response against a variety of pathogens. A large fraction of hematologic malignancies are of B-cell origin, suggesting that the development and activation of B cells are tightly regulated. In recent years, progress has been made in understanding the role of ncRNA in regulating normal B-cell development as well their contribution to disease initiation and progression [5,6,7,8,9,10,11,12,13,14,15,16,17,18,19]. The ability of miRNAs, lncRNAs and circRNAs to influence biological pathways that are dysregulated in disease, in addition to their stability in tissue and biofluids, make them excellent candidates for biomarker discovery [20,21,22,23]. 

Here we provide an overview of the current knowledge on ncRNA expression and molecular functions in normal and malignant B cells, specifically in mantle cell lymphoma (MCL), and discuss their potential clinical value.

## 2. Normal B-Cell Development

B-cell differentiation is a highly regulated process in which uncommitted hematopoietic precursors are differentiated into antibody-secreting plasma cells by a multistep process that begins in primary lymphoid tissue with subsequent functional maturation in secondary lymphoid tissue (human lymph nodes and spleen) [24,25,26,27]. 

**Figure 1:**
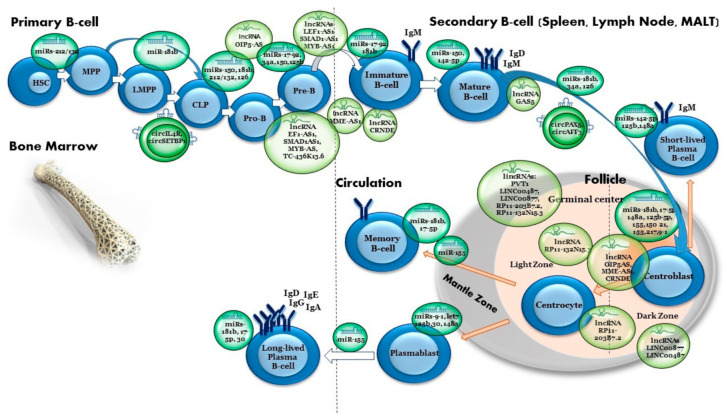
The role of ncRNAs in B-cell development. Normal B-cell development. B-cell development is a stepwise process initiated from hematopoietic stem cells (HSCs) in the bone marrow that develops with multipotent progenitors (MPPs) and further to lymphoid multipotent progenitors (LMPPs) and common lymphoid progenitors (CLPs). At the pro-B- and pre-B-cell stages, the genes encoding the immunoglobulin heavy and light chains of a B-cell receptor (BCR) undergo V(D)J recombination. From the mature B-cell stage onwards, each B cell expresses a unique and functional BCR. Further maturation of B cells occurs in the spleen and gives rise to follicular B cells or a marginal zone. Upon stimulation by cognate antigen, B cells enter the germinal center (GC) where they proliferate and become plasma B cells or memory B cells. Regulation by ncRNAs molecules is listed throughout the differentiating stages.

Lymphoid cells are differentiated in the bone marrow from primitive pluripotent hematopoietic stem cells (HSCs) within the bone marrow by multiple highly regulated developmental stages (Figure 1). The HSCs give rise to uncommitted hematopoietic precursors, known as multipotent hematopoietic progenitors (MPPs). MPPs undergo a dichotomous lineage restriction into common myeloid progenitors (CMPs) and common lymphoid progenitors (CLPs), which give rise to all lymphoid lineages [28]. The CLP compartment contains cells already committed to the B-cell-differentiation pathway. From these lineages, mature B or T cells originate through a network of transcriptional regulators. Pro-B progenitor cells progress to a pre-B cell stage, followed by naive B cells that migrate from the bone marrow to lymphoid organs, where they are transformed into memory and/or antibody-secreting plasma cells [29,30,31,32]. 

Without tight regulation during B-cell development, cascade processes such as V(D)J recombination, somatic hypermutation (SHM), germinal center transit and clonal selection can contribute to malignant transformation of B cells at each step [33].

## 3. Mantle Cell Lymphoma

MCL is an aggressive and incurable lymphoma that constitutes approximately 7% of all B-cell lymphoma cases. The median age at diagnosis is 68, with a male preference ratio of 4:1 [34,35]. Although MCL responds to upfront chemotherapy, it remains incurable, with poor outcomes among patients with the disease. According to the 2016 World Health Organization (WHO) classification of lymphoid malignancies, MCL is classified into two separate subtypes [36]:

- Classical MCL is usually identified by the presence of immunoglobulin heavy-chain variable region gene (IGHV)-unmutated B cells, and SRY-box transcription factor 11 (SOX11) expression. This MCL type usually involves lymph nodes and extranodal sites;
- Leukemic non-nodal MCL is identified by the presence of IGHV-mutated genes without SOX11 expression. This type typically involves the bone marrow, peripheral blood and spleen and mostly has an indolent presentation.

One of the major genetic hallmarks of MCL is the chromosomal translocation t(11;14)(q13;q32), which manifests in the overexpression of cyclin D1 (CCND1) [37,38].

## 4. MicroRNAs—Powerful Regulators of Gene Expression

MiRNAs are short ncRNAs (19–24 nucleotides) that regulate gene expression in a sequence specific manner [39,40]. MiRNAs can function as oncogenes or tumor-suppressors, depending on their target genes. Therefore, identification of specific miRNA expression profiles in normal and tumor tissues may have diagnostic, prognostic and therapeutic implications [20,41,42,43,44]. The ability to detect miRNAs in biological fluids, such as blood, has led to a great deal of interest in the use of miRNAs as biomarkers in “liquid biopsies” [45,46]. To date, more than 2500 human miRNAs (GRCh38) have been identified [47]. 

## 5. MiRNAs in Normal B-Cell Development

Several studies have elucidated the physiological roles of miRNAs as key regulators of hematopoiesis [48,49,50,51]. As miRNAs were discovered earlier, many more studies have been undertaken on theses regulators in the context of B-cell malignancies as compared with lncRNAs and circRNAs.

Over the past decade, a number of studies have shown that miRNAs directly regulate B-cell differentiation at different stages and are, therefore, critical to normal B-cell development (Table 1, Figure 1). Knockout experiments of components in the global miRNA biosynthetic pathway have shown the involvement of miRNAs in normal hematopoiesis [52,53]. First, it was shown that the generation of pre-B cells and subsequently peripheral B cells was impaired in irradiated mice with Ago2-deleted bone marrow cells, which induced a reduction in miRNA levels [53]. In addition, ablation of Dicer at an early stage of B-cell development blocks cells at the pro-B- to pre-B-cell transition [52], whereas Dicer ablation in antigen-activated B cells results in a severe impairment of antibody response and prevention of germinal-center (GC) B cell, long-lived plasma-cell and memory B-cell formation [54]. Two groups who showed that genetic ablation of encoding enzymes is necessary for miRNAs biogenesis, Drosha [55] and the DGCR8 Microprocessor Complex Subunit (DGCR8) [55,56], yielded similar blocks in early B lymphopoiesis in mice. These studies highlighted the important role of miRNAs during the early stages of B-cell development. In 2004, Chen et al. reported the first evidence of the importance of miRNAs in hematopoiesis differentiation. The researchers found that overexpression of miR-181a in hematopoietic stem cells induced an increase in the number of B-lineage cells in both tissue culture and adult mice after re-implantation in bone marrow [57]. Since the identification of miR-181a was reported, other miRNAs have been demonstrated to play determinant roles in lineage differentiation and are discuss in detail below. 

### 5.1. MiR-150

MiR-150 expression is upregulated in hematopoietic progenitors, reducing the normal quantity of mature B cells [58]. The ectopic expression of miR-150 inhibits the progression of pro-B cells to pre-B cells, in part by inducing pro-B-cell apoptosis [58,59]. This is mediated by a dose-dependent repression of the miR-150 target MYB [58]. Consistent with this, partial loss of MYB leads to a dramatic loss of B cells [59], but uncontrolled expression of MYB leads to the development of lymphoid cancers [60,61]. Thus, miR-150 has a critical role in buffering MYB expression within an appropriate range to prevent aberrant B lymphopoiesis.

### 5.2. MiR-17-92 Cluster 

The miR-17-92 family consists of three miRNA clusters: miR-17-92, miR-106a-363 and miR-106b-25. The genomic organization and mature miRNA sequences of this family are conserved in all vertebrates [62,63]. The miR-17-92 cluster regulates the survival of early B-cell progenitors by repressing the expression of Bim (BCL2-like11), a pro-apoptotic gene at the pro- to pre-B-cell transition [64]. Bim is increased in Dicer knockout mice and could be responsible, at least in part, for the massive apoptosis observed at the pre-B stage. In contrast, the overexpression of this cluster in transgenic mice results in lympho-proliferative and autoimmune phenotypes due to reduced phosphatase and tensin homolog (PTEN) and Bim protein expression [65]. Altogether, these data suggest that the miR-17-92 cluster has a role in the proliferation control of B cells. B-cell development has been examined in triple knockout B-cell mice lacking the miR-17-92 family (miR-17-92, miR-106a-363 and miR-106b-25). A block in B-cell development and increased apoptosis, similar to that observed in Dicer ablation mice, but not Bim upregulation, were observed, suggesting that Bim was not responsible for apoptosis in this model [66]. 

### 5.3. MiR-34a

MiR-34a, which is expressed at low levels in early B cells, regulates B-cell differentiation from pro-B cell to pre-B cell. Ectopic expression of miR-34a confirmed the existence of a blockage at the pro-B-cell stage by downregulation of Forkhead box P1 (Foxp1) protein, a transcriptional regulator required for B-cell differentiation [67] which is a direct target of miR-34a. Knockdown of miR-34a resulted in increased amounts of Foxp1 and mature B-cells [68].

### 5.4. MiR-126

Okuyama et al. (2013) suggest that miR-126 has a crucial role in B-cell lineage commitment. Overexpression of miR-126 in an immature B-cell line or in mouse lin-HSPC induced B-cell differentiation through the regulation of insulin regulatory subunit1 (IRS1) [69]. 

### 5.5. MiR-212/132 Cluster 

The miR-212/132 cluster has emerged as an important regulator of HSC function. This cluster is enriched in HSCs and is upregulated during aging. Both overexpression and deletion of miRNAs in this cluster lead to inappropriate hematopoiesis with age. Enforced expression of miR-132 in the bone marrow of mice led to rapid HSC cycling and depletion. In addition, miR-132 exerted its effect on aging HSCs by targeting the transcription factor FOXO3, a known aging-associated gene [70].

The cluster has also been shown to inhibit the pre-pro-B- to pro-B-cell transition by targeting SOX4 [71], a crucial mediator of pro-B-cell development. The miR-212 and miR-132 clusters establish a checkpoint for B-cell development by setting a threshold for the expression of SOX4. In order for B-cell development to continue, the expression of the target must exceed a certain concentration to bypass miRNA-mediated negative feedback. MiR-212/132 is also deregulated in certain B-cell cancers [72,73,74].

## 6. Germinal Center Regulation by microRNAs

The latter stages of B-cell development involve the activation of naive B cells and the formation of plasma cells [75]. The GC reaction involves the clonal expansion of antigen-specific B-lymphocytes and the generation of B cell subclones with related antigen specificities, from which subclones expressing immunoglobulins with improved affinity for the antigen are positively selected. Within the GC, proliferating GCB cells undergo SHM, affinity maturation and the class switch recombination (CSR) reaction and finally differentiate into memory B cells or plasma cells [76]. This path is also regulated by several specific miRNAs as described below (Figure 1).

### 6.1. MiR-155 and 181b 

These two miRNAs were shown to target the activation-induced cytidine deaminase (AID) mRNA [77,78]. AID expression is required during the B-cell SHM process of immunoglobulin genes and its loss leads to the impairment of class switching [79]. Therefore, expression of miR-181b and miR-155 impairs SHM and CSR, which is necessary for antibody maturation. Additionally, deregulated AID expression can lead to hematopoietic cancers [80]. The importance of miR-155 in GC regulation has been further documented by the fact that miR-155 negatively regulates the expression of CD10, a marker of GC cells [81]. Moreover, miR-155 also regulates specific B-cell subpopulation maturation in mice, where loss of miR-155 leads to a reduction in IgG1 switching by targeting the PU.1 transcription factor [82], which, in turn, leads to PAX5 downregulation, enabling plasma cell commitment [83].

The causal role of miR-155 in lymphoma has been established in two mouse model studies. Functional studies of miR-155 in anaplastic large cell lymphoma have demonstrated its association with lymphocyte differentiation and immunity [84]. The biological information databases TargetScan and miRdb predict that miR-155 may participate in the regulation of sex-determining region Y-box transcription factor 11 (SOX11), a definitive diagnostic indicator of MCL. Furthermore, elevated expression of miR-155 is associated with the transformation of monoclonal B lymphocytosis (MBL) to chronic lymphocytic leukemia (CLL), poor therapeutic response and short OS in these patients [85]. 

### 6.2. MiR-125b 

Another miRNA with a role in the final step of B-cell maturation in mice is miR-125b, which represses both PR/SET domain 1 (PRDM1; also known as BLIMP1) and tinterferon regulatory factor 4 (IRF4) transcription factors, essential for plasma cell differentiation. Overexpression of miR-125b in an LPS-responsive B cell resulted in inhibition of PRDM1 expression and IgM secretion. Overexpression of this miRNA also inhibited the differentiation of primary B cells and compromised the survival of cultured myeloma cells. These findings suggest that miR-125b promotes B lymphocyte diversification in the GC by inhibiting premature utilization of essential transcription factors for plasma cell differentiation [86].

### 6.3. MiR-9, MiR-30 and Let-7 Families 

Among various transcriptional regulators, B-cell lymphoma 6 (BCL6) and PRDM1 are master regulators for GC formation and terminal B-cell differentiation. Dysregulation of BCL6 and PRDM1 has been associated with lymphomagenesis [87,88]. Follicular dendritic cells (FDCs), constituting the backbone of follicles, are a major component of the follicular microenvironment and have an anti-apoptotic role in B-cell survival and late B-cell development.

Lin et al. (2011) have shown direct cell–cell contact between FDCs and B-lymphocytes by influencing the expression of a set of miRNAs regulating the expression of BCL6 and PRDM1. FDC induced upregulation of PRDM1 expression through downregulation of the miR-9 and let-7 families and induced downregulation of BCL6 through upregulation of the miR-30 family in B-lymphocytes and lymphoma cells. Furthermore, they demonstrated that the miR-30 family directly controls BCL6 expression and that miR-9-1 and let-7a directly control PRDM1 expression by targeting their 3′UTR, mediating the effect of FDC. The study revealed a novel regulatory mechanism in which FDC, through induction of miRNAs in B-lymphocytes, affects the regulation of transcription factors, promoting GCB cell survival and differentiation. Dysregulation of miRNAs may interfere with B cell survival and maturation, thus representing a novel molecular mechanism, as well as a potential therapeutic target in B-cell lymphomas [89].

### 6.4. MiR-21

Interleukin (IL)-21 signaling is an important regulator of plasma cell differentiation initiation. This signal activates signal transducer and activator of transcription 3 (STAT3) and IRF4, leading to the induction of both PRDM1 and miR-21. MiR-21 is repressed during IL-21-driven plasma cell differentiation. The molecular basis for this repression was the identification of a primary miR-21 transcript as a direct target of PRDM1-dependent repression, despite continued STAT3 activation and phospho-STAT3 binding to the primary miR-21 promoter. Thus, this miRNA participates in an incoherent feed-forward loop that allows for early expansion of cells that are committed to the plasma cell fate, with a temporally delayed repression of this effect to avoid deregulated proliferation [90].

### 6.5. MiR-217 

MiR-217 was identified as a positive regulator of the GC reaction and as an oncogene promoting mature B-cell lymphomagenesis. Overexpression of this miRNA boosted the number of GC B cells and promoted SHM and CSR reactions. Conversely, inhibition of endogenous miR-217 limited these events. Interestingly, miR-217 gain of function did not promote any measurable alterations in B-cell differentiation, suggesting that the function of miR-217 in the B-cell lineage is restricted to the context of GCs and antibody variation.

MiR-217 also downregulated the expression of a DNA damage response and repair gene network and in turn stabilized BCL6 expression in GC B cells. Importantly, miR-217 overexpression also promoted mature B-cell lymphomagenesis. This is physiologically relevant since miR-217 was found to be overexpressed in aggressive human B-cell lymphomas. Therefore, miR-217 provides a novel molecular link between the normal GC response and B-cell transformation [91].

### 6.6. MiR-142 

MiR-142 is abundantly expressed in immune cells. MiR-142 knockout mice revealed that this miRNA has a critical role in the development and homeostasis of lymphocytes; marginal zone B cells expanded in the knockout spleen, whereas the numbers of T and B1 cells in the periphery were reduced. This abnormal development of hematopoietic lineages in the knockout mice was accompanied by a profound immunodeficiency, manifested by hypogammaglobulinemia and failure to mount a productive immune response to soluble antigens and virus. 

B cells with miR-142 knockout express elevated levels of B-cell-activating factor receptor (BAFF-R), proliferating more robustly in response to BAFF stimulation. Lowering the BAFF-R gene dose in miR-142 knockout mice recuperated the B-cell expansion defect, suggesting that BAFF-R is an miR-142 target through which it controls B-cell homeostasis. Overall, these findings proved that miR-142 as an essential regulator of lymphopoiesis and suggested that lesions in the miR-142 gene may lead to primary immunodeficiency [92].

### 6.7. MiR-148a 

Porstner et al. (2015) proposed that miR-148a is a new player in the regulatory network controlling terminal plasma cells differentiation. Transcription factors such as BTB domain and CNC homolog 2 (Bach2) and melanocyte-inducing transcription factor (Mitf) delay premature differentiation of GC B cells by repressing PRDM1and IRF4 (required for terminal plasma cell differentiation). MiR-148a is the most abundant miRNA in primary human and murine plasma cells, and its expression is upregulated in activated murine B cells and overlaps with PRDM1 synthesis. Furthermore, miR-148a targets Bach2, Mitf and proapoptotic factors such as PTEN and Bim [93]. Elevated expression of miR-148 was also shown by another group to impair B-cell tolerance by targeting growth arrest and DNA damage-inducible alpha (GADD45α), an autoimmunity suppressor implicated in mitogen-activated protein kinase (MAPK) pathway regulation [94]. The authors concluded that miR-148a might be crucial for the regulation of apoptosis in immature B cells upon BCR activation and that miR-148a might regulate B-cell survival by targeting Bim, PTEN and GADD45α.

## 7. MiRNAs Related to MCL

**Figure 2:**
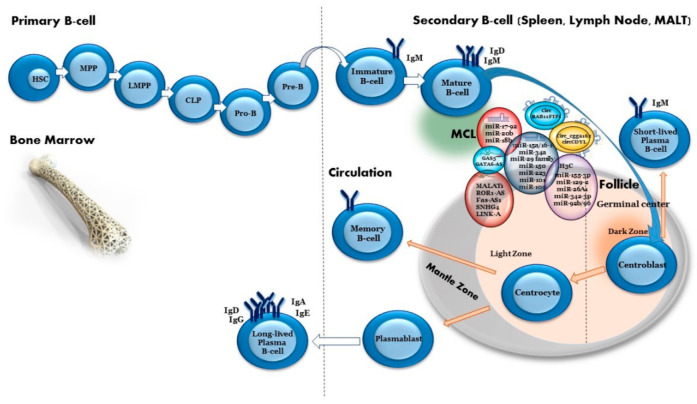
The role of ncRNAs in mantle cell lymphoma.

Several studies investigated the expression and function of miRNAs in MCL using different biological models (cell lines, patient samples and an animal model). Schraders et al. (2008) combined an analysis of tiling-resolution array-CGH with gene expression profiling of 11 MCL tumors, which enabled the identification of genomic alterations and their corresponding gene expression profiles and the identification of several genomic regions harboring miRNAs that were gained or lost in MCL [95]. Since then, several groups have identified deregulated miRNAs, with both biological and prognostic implications for MCL (Table 2, Figure 2).

### 7.1. The miRNA-17~92 Cluster

Navarro et al. (2009) investigated the expression of 86 mature miRNAs, mapped to frequently altered genomic regions in MCL, in CD5(+)/CD5(−) normal B cells, reactive lymph nodes and purified tumor cells of leukemic MCL, nodal MCL and MCL cell lines [96]. The miRNA-17~92 cluster was upregulated in both MCL lymph nodes and leukemic MCL cells. This observation was supported by a later study [30], which demonstrated that high expression of the transcript C13orf25 (which is the host mRNA of miRNA-17~92 cluster) was associated with shorter median overall survival (OS) in a small group of patients (1.06 vs. 2.75 years). 

The miRNA-17~92 cluster has been associated with cell proliferation. This was shown by a genome-wide miRNA profiling study. Samples from MCL patients were classified into three groups based on mRNA proliferation signatures. High proliferation signatures were correlated with a significantly upregulated miRNA-17~92 cluster, indicating an association between these clusters and proliferation [97]. Deshpande et al. (2009) showed that CCND1 3’UTR contain miRNA-17~92 binding sites in MCL [98]. In addition, overexpression of these cluster members was correlated with high MYC expression in aggressive MCL [96]. Likewise, MCL cell lines demonstrated a high proliferation gene signature, activation of the PI3K/AKT pathway, as well as inhibition of chemotherapy-induced apoptosis [99]. Rao et al. (2012) demonstrated that PH domain and leucine-rich repeat protein phosphatase 2 (PHLPP2), a key regulator of the PI3K/Akt pathway, is targeted by the miRNA-17~92 cluster along with PTEN and BIM. Furthermore, they demonstrated that inhibition of miRNA-17~92 expression in a xenograft MCL mouse model inhibited the PI3K/Akt pathway, decreasing tumor growth. Given the insufficient efficacy of standard treatments in poor risk patients with MCL, novel therapeutic approaches that target the miR-17-92 cluster appear to be an attractive option for MCL patients [99]. Similarly, Jiang et al. (2010) demonstrated that the overexpression of miR-17-92 significantly increased the radio-resistance of human MCL cells via the PI3K/AKT pathway by targeting PTEN and PHLPP2. They showed that in human MCL cells, the miR-17-92 cluster is overexpressed after different radiation doses, leading to the enhancement of AKT serine/threonine kinase activity and the significant increase of cell survival and cell proliferation and decrease of cell death. This finding suggested miR-17-92 as novel target molecule to enhance radiotherapy sensitivity of MCL in the clinic [100]. Finally, Roisman et al. (2016) showed that differential expression profiles of SOXC and miR-17~92 family miRNAs could discriminate between the different clinical subtypes of MCL [101]. 

### 7.2. The miR-16-1/miR-15a Cluster

The direct role of miRNA deregulation in cancer biology was first described in B cell malignancies when miR-15a and miR-16-1 were found in the 13q14 locus, which is frequently deleted in CLL [102].

As mentioned above, one of the major hallmarks of MCL is the t(11:14) translocation, which leads to overexpression of CCND1 [37,38]. In some patients, truncations within the CCND1 mRNA 3’UTR result in a worse prognosis. Chen et al. (2008) have shown that miR-16-1 regulates CCND1 protein expression by demonstrating that the two binding sites for miR-16-1, which are normally located in the 3′UTR of CCND1 mRNA, are lacking in the truncated form, preventing proper miR-16-1 regulation of CCND1. The absence of miR-16-1 binding to the truncated CCND1 can partly explain its prolonged mRNA viability and increased cell cycle progression. However, the loss of AU-rich elements in the 3′UTR of CCND1 also contributes to prolonged mRNA stability [103]. 

Another study (2010) found that the miR-16-1/miR-15a cluster is downregulated in MCL patient samples [104]. Further experimental studies showed that this is likely caused by the oncoprotein MYC, which, through interaction with histone deacetylase 3 (HDAC3), represses the expression of these two miRNAs [105]. Teshima et al. (2014) showed that miR-16 targets the proto-oncogene Bmi1, which is overexpressed in various types of tumors, particularly in aggressive tumors and in tumors resistant to conventional chemotherapy [106]. Bmi1 is also crucially involved in cancer-initiating cell maintenance [107,108,109,110,111] and was found to be upregulated in recurrent MCL [106]. The researchers showed that miR-16 downregulated Bmi1 expression, leading to reductions in tumor size following lymphoma xenografts. In addition, they demonstrated that Bmi1 directly regulated pro-apoptotic genes such as BCL2L11/Bim and phorbol-12-myristate-13-acetate-induced protein 1 (PMAIP1)/Noxa, leading to an enhanced anti-apoptotic potential of MCL. Finally, bortezomib, a proteasome inhibitor used for treating relapsed MCL, effectively induced apoptosis among MCL cells while reducing the expression of Bmi1 and increasing miR-16 levels. Based on these results the authors suggested that targeting Bmi1 might be an effective approach to treating refractory and recurrent MCL [106]. Finally, miR-15b was shown to play a role in MCL transformation to an aggressive disease [112].

### 7.3. MiR-34a 

MiR-34a was the first miRNA implicated in DNA damage response, as it is transactivated by p53 [113]. This miRNA controls several genes that are associated with MCL pathogenesis, including cyclin-dependent kinase (CDK)-4, CDK6, MYC and CCND1 [114]. Navarro et al. (2013) categorized high and low expression levels of miR-34a in 30 leukemic MCL cases. Low expression of miR-34a was associated with a significantly shorter OS (*p* < 0.001) in patients with leukemic and nodal MCL who also had high MYC expression (median OS for leukemic and nodal cases: 9 and 21 months, respectively) in comparison to patients with only one of these factors (median OS for leukemic and nodal: 58 and 49 months, respectively) or none of them (median OS for leukemic and nodal: not reached and 64 months, respectively). These results indicate that downregulation of miR-34a together with MYC overexpression contribute to the aggressiveness of MCL [115].

Moreover, loss of miR-34a was associated with worse prognosis (median OS, 25 months) compared to patients with higher miR-34a expression (median OS, 67 months). In addition, miR-34a targets the oncogene MYC, whose overexpression has been associated with poor outcomes in MCL [116,117]. 

### 7.4. MiR-29 

MiR-29b has been associated with various disorders including fibrotic diseases, cancers, and neurodegenerative diseases [118,119]. 

Reduced miR-29 expression has a potential prognostic value in predicting the course of MCL. Zhao et al. (2010) have identified the miRNA expression signature and frequent deregulation of a set of miRNAs (miR-150, miR-142-3p/5p, miR-29a/b/c, miR-124a and miR-155) in MCL [104]. It was suggested that downregulation of miR-29 could be a potential molecular marker for discriminating MCL prognosis. Specifically, MCL patients with significantly downregulated miR-29 levels demonstrated shorter OS compared with those who expressed relatively high levels of miR-29. Furthermore, inhibition of miR-29 was demonstrated to activate CDK4/CDK6 in MCL as well as phosphorylation of retinoblastoma-associated protein RB1. Hence, miR-29 may also serve as a therapeutic target for MCL intervention. 

### 7.5. MiR-150 

MiR-150 levels are consistently low in MCL [120,121]. Zhang et al. (2020) have found that MiR-150 inhibits the proliferation and promotes the apoptosis of MCL cells by negatively regulating MET expression. Upregulation of miR-150 significantly suppressed the proliferation of primary MCL cells. Increased expression of MET remarkably facilitated the apoptosis of primary MCL cells. Therefore, miR-150 mimicking may be used as therapeutic agent due to a strong association of low miR-150 levels with worse prognosis, active BCR signaling and B-cell proliferation [122]. 

### 7.6. MiR-18b 

MiR-18b expression is an important marker of cell proliferation and cell adhesion in hepatocellular carcinoma (HCC) and may be used as a diagnostic and prognostic marker for HCC progression [123,124]. However, miR-18b decreased the proliferation rate without inducing apoptosis in MCL patients, suggesting that it may render MCL cells resistant to chemotherapy by decelerating cell proliferation. MiR-18b overexpression is associated with poor outcome of MCL, improving the MIPI-B prognosticator. Husby et al. (2015) confirmed the prognostic and predictive value of miR-18b expression disorders and proposed introducing it into the new biological MCL International Prognostic Index (MIPI-B)-miR prognosticator, combining expression levels of miR-18b with MIPI-B data [123].

### 7.7. MiR-20b 

High levels of miR-20b expression have been associated with worse prognosis of several types of cancers [125,126]. In MCL, lack of miR-20b expression had a survival probability of 56% at 60 months, whereas only 33% of patients included in the high-risk group (high level of expression of miR-20b) survived for 460 months [121]. Szymczyk et al. (2018) confirmed that overexpression of miR-10a, miR-20b and miR-363 translates into OS reduction [126]. MiR-127-3p and miR-146a expression may also have a potential prognostic value [97,126].

### 7.8. MiR-223 

MiR-223 is a hematopoietic-specific miRNA with crucial functions in myeloid lineage development [127,128]. MiR-223 was downregulated in purified CD19+ lymphocytes from MCL patients compared to healthy individuals. In addition, low miR-223 expression predicted poor OS of MCL patients regardless of treatment. Furthermore, overexpression of miR-223 in an MCL cell line inhibited cell proliferation and promoted G0/G1 accumulation and cell apoptosis. The researchers confirmed by luciferase reporter assay that miR-223 suppressed the wild-type 3′UTR of SOX11, a crucial transcription factor in MCL that was found to be negatively correlated with the mRNA level of SOX11 in clinical samples [129,130]. 

### 7.9. MiR-101 

MiR-101 is downregulated in MCL as compared with a control group and an enhancer of the zeste 2 polycomb repressive complex 2 subunit (EZH2) protein, which is highly expressed in MCL. A negative correlation was found between miR-101 and EZH2 expression, which was significantly correlated with B symptoms, International Prognostic Index and Ann Arbor stage. The OS rate of patients with low miR-101 expression was significantly lower than that of patients with high miR-101 expression, and the OS rate of patients with high EZH2 expression was significantly lower than that of patients with low EZH2 expression. Transfection of MCL cells with a miR-mimic downregulated the EZH2 protein, inhibited the proliferation of MCL cells and increased their apoptosis rate [131].

### 7.10. MiR-100 

MiR-100 can be a tumor suppressor gene or oncogene, and its expression and function vary in different tumors and sometimes have opposing roles. However, in the case of MCL, it was reported to be associated with inhibition of cell proliferation and promotion of apoptosis by targeting mTOR in both MCL tissues and cell lines [132].

## 8. Methylation of miRNA Genes in MCL 

Epigenetic mechanisms such as DNA methylation allow for a precise gene expression cascade, which is programmed and shaped by transcription factor binding and interaction between DNA methyltransferases and histone marks, which is needed during cellular differentiation. However, these mechanisms become deregulated during tumorigenesis and have been shown to contribute to the pathogenesis of both solid and hematologic malignancies [133,134,135].

### 8.1. MiR-155-3p

Methylation-specific polymerase chain reactions (PCRs) in MCL cell lines and in primary patient samples verified that miR-155-3p undergoes complete methylation, resulting in upregulation of lymphotoxin-beta (LT-β), which is a direct target of miR-155-3p. Since LT-β is a positive regulator of non-canonical NF-κB signaling, miR-155-3p methylation is implicated in lymphomagenesis. Treatment of MCL cell lines with 5-aza-2′-deoxycytidine resulted in demethylation and re-expression of miR-155-3p. Overexpression of miR-155-3p led to increased sub-G1 apoptotic cells and reduced cellular viability, demonstrating its tumor suppressive properties [136].

### 8.2. MiR-129-2

Epigenetic inactivation of miR-129-2 was studied by methylation-specific PCR in 13 cell lines, which included 5 lymphoma and 8 myeloma cell lines. All five lymphoma and seven of the eight myeloma cell lines showed complete and partial miR129-2 methylation. Hypomethylation treatment of the MCL cell-line, JEKO-1, homozygously methylated for miR129-2, led to miR129-2 demethylation and miR129 re-expression. MiR-129 overexpression in both mantle cell lines, JEKO-1 and GRANTA-519, inhibited cellular proliferation and enhanced cell death, with concomitant SOX4 mRNA downregulation [137].

### 8.3. MiR-26A1 

In a study published in 2016, the authors observed that miR-26A1 is uniformly hypermethylated in 24 MCL patients. Extended analysis using pyrosequencing confirmed the findings and real-time quantitative PCR verified low miR-26A1 expression in both CLL and MCL compared to normal B cells. The levels of EZH2, a known target of miR-26a, were reduced by overexpression of miR-26A1 in CLL and MCL cell lines. In addition, treatment with a methyl-inhibitor resulted in miR-26A1 upregulation with a parallel decrease in EZH2 expression. Finally, increased apoptosis was observed in cell lines overexpressing miR-26A1, further underscoring the functional relevance of miR-26A1 [138].

### 8.4. MiR-342-3p 

MiR-342-3p, which is localized to chromosome 14q32, is a tumor suppressor co-regulated with its host gene Enah/Vasp-Like (EVL). Bisulfite pyrosequencing verified by methylation-specific PCR enabled detection of EVL/MIR342 methylation in lymphoma cell lines but not in normal peripheral blood and tonsils. Promoter demethylation treatment resulted in re-expression of miR-342-3p and EVL. The tumor suppressor function of miR-342-3p was demonstrated by the inhibition of cellular proliferation and increased cell death [139]. 

### 8.5. MiR-92b/96 

Aberrant expression of miR-92b and miR-96 is associated with enhanced protein arginine methyltransferase-5 (PRMT5) translation, which is overexpressed in aggressive B-cell non-Hodgkin’s lymphomas, including MCL and diffuse large B-cell lymphoma (DLBCL). PRMT5 supports the constitutive expression of CCND1 and c-MYC, interacts with human switch/sucrose non-fermentable (SWI/SNF) complexes and methylates histones H3R8 and H4R3 [140,141]. Re-expression of miR-92b and miR-96 inhibited PRMT5 translation in vivo. PRMT5 knockdown altered the growth characteristics of MCL cell lines.

## 9. Long Non-Coding RNAs in Normal Development

LncRNAs have a wide functional range, with some well-described mechanisms, including modulation of chromatin structure [142,143], protein scaffolding functions [144,145,146,147] and transcriptional and epigenetic regulation through interactions with DNA, RNA and proteins [10]. 

Only a few lncRNAs show high sequence conservation across species, among them XIST, PVT1, MIAT, NEAT1, MALAT1 and OIP5-AS [148]. The biological significance of lncRNAs was proven in a murine knockout study in which developmental defects and lethality occurred upon deletion of several lncRNAs [149]. Large-scale screening of 700 lncRNA knockouts in human cell lines identified that 50 of them have a significant effect on cancer cell growth [150]. Thus, although the functional significance of the bulk of lncRNAs is unclear, a significant proportion of them have major biological functions.

Several studies have revealed the involvement of lncRNAs in different stages of human B-cell development [10,151,152,153,154,155,156,157]. Casero et al. (2015) have identified global lncRNA expression patterns that corresponded to early (progenitor stage) lymphoid commitment or lineage (B or T) specification in bone marrow, indicating their importance in lineage commitment. This expression pattern was highly stage specific and more lineage-specific than that observed for protein-coding genes. Protein-coding genes co-expressed with neighboring lncRNA genes showed enrichment for ontologies related to lymphoid differentiation. The global lncRNA expression patterns revealed developmental relationships among the earliest progenitor cells in the human bone marrow and thymus [152].

At later stages of B-cell development and maturation, lncRNA expression profiles can be highly similar between functionally distinct B cells, such as follicular and marginal zone B cells in the spleen [156] and naive and memory cells in tonsils [155]. However, the strongly proliferative GCB cells showed an lncRNA profile that is very distinctive from other mature B-cell subsets [152,155,156]. Petri et al. (2015) identified early B-cell development-specific genes, expressed in pre-BI, pre-BII and immature B cells, such as recombination activating 2 (RAG2), v-set pre-b cell surrogate light chain 1 (VPREB1), DNA nucleotidylexotransferase (DNTT), lymphoid enhancer binding factor 1 (LEF1), SMAD1 and MYB, which were associated with the antisense transcripts LEF1-AS1, SMAD1-AS1 and MYB-AS1, as well as with the intergenic transcript CTC-436K13.6 [153]. Mitotic cell cycle-related genes such as kinesin family member 23 (KIF23), polo-like kinase 4 (PLK4) and centromere protein E (CENPE) were specific for the proliferative stages of B-cell development, specifically pre-BI, pre-BII, centroblasts and centrocytes, and were associated with the lncRNAs OIP5-AS and MME-AS1 and the bidirectional lncRNA CRNDE. CRNDE has previously been linked to cell cycle and proliferation [158,159,160,161]. Expression of AID and serpin family a member 9 (SERPINA9), two genes specifically expressed in GC centroblasts and centrocytes, was associated with PVT1 and multiple uncharacterized lincRNAs, such as LINC00487, LINC00877, RP11-203B7.2 and RP11-132N15.3. The latter is located 240 kb upstream of the BCL6 transcription repressor. RNA-seq of 11 murine B-cell subsets revealed 4516 differentially expressed lncRNAs [156]. Assessment of the histone H3K4 mono/trimethylation ratio of these differentially expressed lncRNAs revealed 192 promoter (high H3K4me3)- and 702 enhancer (high H3K4me1)-associated lncRNAs (eRNAs). Comparison with previous human studies [151,152,153] identified 228 eRNAs with a potential human ortholog based on positional conservation and 185 based on sequence conservation. Notably, the abovementioned GCB cell-associated lncRNA RP11-132N15.3, located downstream of BCL6 [153], has a murine ortholog showing both sequence and positional conservation. However, this lncRNA appears to be downregulated in murine GCB cells [156], while it is upregulated in human GCB cells, indicating that despite strong similarities a functional conservation is unlikely. Thus, although most current studies are limited due to the inclusion of a restricted number of B-cell subsets [151,152,153,155] or the use of microarrays preventing the identification of novel transcripts [153,155], they provide a valuable overview of B-cell subset-specific lncRNAs. 

## 10. Long Non-Coding RNA in MCL

According to the lncRNA profile in MCL, determined by next generation RNA sequencing, ROR1-AS1 (also called RP11-24J), which is predominantly localized in the nucleus, was significantly upregulated in most MCL tumor samples and MCL cell lines (Mino, Granta, JVM2 and Z138) compared to non-tumor controls. Overexpression of ROR1-AS1 lncRNA promoted growth of MCL cells and resistance to ibrutinib (BTK inhibitor) and dexamethasone treatment through regulation of SOX11 and P16 expression [162]. The increased levels of ROR1-AS1 lncRNA may have been a result of translation upregulation of EZH2, a component of the polycomb repressive complex 2 (PRC2), through lncRNA MALAT1. MALAT1 is a well-established oncogene which is strongly associated with cancer progression by repression of the TP53 promoter. MALAT1 increases EZH2 translation. In turn, EZH2 binds a ROR1-AS1 lncRNA, which increases cell proliferation in MCL cell lines [163,164]. In addition, the promoter region of FAS-AS1 is also regulated by EZH2. This lncRNA serves to modulate alternative splicing of the FAS gene, a critical molecule in the extrinsic apoptosis pathway. FAS-AS1 leads to decreased exon skipping and upregulation of the membrane-bound FAS isoform, whereas the soluble isoform (sFAS) that inhibits apoptosis is downregulated [165]. 

Treatment with DzNep (EZH2 methyltransferase inhibitor) or ibrutinib increased FAS ligand-mediated apoptosis in lymphoma cell lines by abolishing the EZH2-mediated repression of FAS-AS1 expression, resulting in decreased expression of sFAS [7].

In another study, lncRNAs associated with translation machinery were identified in tumor cells of MCL patients by RNA immunoprecipitation (RIP) sequencing. Translation machinery usually regulates the translation of mRNAs via translation initiation factor-4E ([eIF4E], a key component of the translation initiation complex), whose overexpression in MCL tissue was correlated with poor prognosis [166]. A further study reported that eIF4E is dysregulated and small nucleolar RNA host gene (SNHG) 4 is overexpressed in MCL cell lines. The lncRNAs, SNHG1 and SNHG4 can bind eIF4E and regulate protein translation. The authors also suggested that SNHG4 lncRNAs might serve as potential biomarkers for MCL and other B-cell lymphomas for translation therapy [167]. Another study has revealed the top eight eIF4E-enriched lncRNAs in MCL patient samples as compared with normal controls, including novel (NBPF8 and RP4-550H1.6) and known lncRNAs (ZNFX1-AS1, SNHG5, FTX, GAS5, CECR7 and SNHG12). Among them, SNHG12, FTX, SNHG5 and ZNFX1-AS1 were found to be associated with translation machinery via eIF4E-RNA-binding motifs (but not the cap-binding domain) in MCL tumor cells. SNHG5 and SNHG12 can also modulate c-Myc translation in MCL cells [168].

In addition, the GAS5 lncRNAs, a proposed tumor-suppressor, interacted with c-Myc mRNA and reduced its translation. Knockdown of GAS5 resulted in decreased apoptosis levels, and a treatment effect of mTOR inhibitors in MCL cell lines [169].

LncRNA GATA6 antisense (GATA6-AS) was also shown to be involved in endothelial–mesenchymal transition and therefore may play a critical role in the progression of MCL. Overexpression of lncRNA GATA6-AS downregulated glucose transporter 1 (GLUT1), thereby inhibiting glucose uptake and resulting in reduced cell proliferation. Plasma lncRNA GATA6-AS expression levels were downregulated in patients with MCL compared with those in healthy controls. These findings suggest that lncRNA GATA6-AS has potential diagnostic value in early stage MCL in addition to inhibiting cancer cell proliferation through its overexpression [170].

The LINK-A lncRNA was also investigated in MCL. This lncRNA acts as an oncogene in triple-negative breast cancer. Plasma levels of LINK-A lncRNA and survivin, a known member of the inhibitor of apoptosis (IAP) family, were significantly increased in patients with MCL in comparison to healthy controls. LINK-A lncRNA overexpression promoted cell proliferation, inhibited cell apoptosis and upregulated survivin expression, while its knockdown showed the opposite effect [171]. These findings suggest that LINK-A lncRNA is an oncogene in MCL and indicate a potential application in the diagnosis of the disease. 

## 11. Circular RNAs 

Among the large class of lncRNAs, a newly recognized subclass, named circular RNA (circRNA), is particularly interesting. Thousands of endogenous circRNAs in mammalian cells have been discovered, some of which are highly abundant and evolutionarily conserved [172].

Unlike linear RNAs, circRNAs are formed as a circular structure by joining the 3′ end of the RNA to the 5′ end. Due to the lack of free ends, they are very stable molecules for exonuclease cleavage and thus they have great potential as innovative therapeutic approaches and are suitable biomarkers for diagnosis and disease progression [7].

Some of the circRNAs co-sediment with ribosomes, indicating their translation potential, but a vast majority of these molecules act as molecular decoys for miRNAs or RNA-binding proteins (RBPs) and so-called miRNA sponges.

CircRNAs are classified into three types according to their sequences: exonic (ecircRNAs), composed of only exons and mainly found in the cytoplasm; intronic (ciRNAs), composed of only introns and predominantly detected in the nucleus; and exon-intronic (EIciRNAs), composed of introns at the region between exons and mainly located in the nucleus [173]. 

CircRNAs are abundant in the nucleus, bind to RNAs and regulate selective splicing or promote transcription [174,175].

## 12. Circular RNA in B-Cell Development and Malignancies

Several studies have shown that circRNAs are involved in normal B-cell differentiation and lymphoid tissue development, and that they have an important role in the pathogenesis of various diseases including cancer. However, only a few studies have examined their role as drivers of carcinogenesis in B-cell malignancies including MCL [7,176,177]. 

In some hematological malignancies, chromosomal translocations can give rise to fusion-circRNAs. For example, two circRNAs transcribed from the MLL-AF9 translocation observed in ALL have oncogenic properties [178]. It is still unknown whether chromosomal translocations in other B-cell malignancies also give rise to fusion-circRNAs.

In Burkitt lymphoma, which is characterized by high MYC expression, two circRNAs, ZDHHC11 and ZDNN11B, containing multiple binding sites for miR-150 were upregulated following upregulation of the MYC that targets MYB. ZDHHC11 and ZDNN11B act as endogenous sponges that bind to miR-150. When a panel of BLBCL, Hodgkin and Burkitt lymphoma cell lines were compared to GCB cells it was revealed that MYC, ZDHHC11/B and MYB were upregulated and miR-150 was downregulated in all lymphoma types as compared to GCB [179]. As described before, miR-150, which is regulated in cell lines expressing high levels of MYC, is an important regulator of hematopoiesis that strongly inhibits cell growth and is proposed to be a tumor suppressor. Another study showed that a low level of circRAB11FIP1, which originates from exon 2 of the host gene RAB11-family interacting protein 1, was significantly associated with shorter time to progression and the presence of TP53 mutations. To date, additional studies are needed to examine whether there is a functional link between circRAB11FIP1 and TP53 in MCL, which may help explain the poor prognosis in patients with TP53 mutations [180].

High-throughput RNA sequencing of several MCL cell lines (REC-1, Granta-519, UPN2, Z138) used to profile the genome-wide landscape of circRNA expression detected several circRNAs which have previously been implicated in cancer, including ciRS-7, circHIPK3, circCCDC66, circFBXW7, circSMARCA5, circCDYL and circZKSCAN1, as well as a novel circRNA from the IKZF3 gene that has been previously linked to MM [181]. These findings suggest that circRNAs may play important roles in the pathogenesis of MCL.

CircCDYL was reported to be clinically significantly upregulated in MCL [182]. This circRNA, derived from exon 4 of the CDYL gene, is a histone methyllysine reader and transcriptional corepressor through back-splicing [183]. CircCDYL was highly expressed in the plasma of MCL patients compared to healthy donors and its knockdown inhibited MCL cell proliferation. These findings indicate that circCDYL might serve as a potential diagnostic biomarker in clinical practice or as an additional biomarker to increase the diagnostic accuracy of MCL [182].

Li et al. (2020) reported that the expression level of Circ_cgga162 in MCL patients was significantly higher than that of the control group and was correlated with the MCL International Prognostic Index (MIPI) score, Ann Arbor stage, bone marrow infiltration and Ki67, but not with age, gender, B symptoms or LDH. These findings suggest that Circ_cgga162 can be used as a potential prognostic marker and therapeutic target in MCL patients [184].

**Figure 3:**
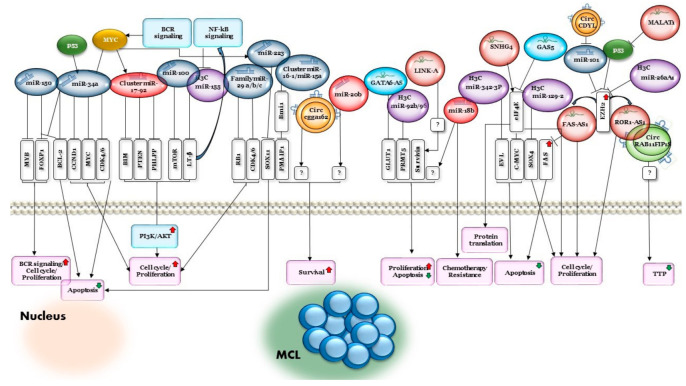
Biological pathways regulated by ncRNAs in MCL.

A summary of the biological pathways regulated by ncRNAs can be found in Figure 3.

## 13. Conclusions 

Despite numerous developments, gene networks regulated by ncRNAs in B cells requires further investigation. The majority of published papers only examined the role of a specific miRNA, lncRNA or circRNA in the regulation of a few genes, selected based on target prediction or a well-known function in lymphomas, irrespective of the context of other ncRNAs in the cell. Continued investigation of ncRNA biogenesis and function will be necessary to examine the potential use of ncRNAs as predicting and diagnostic markers and as therapeutic tools. Moreover, translational therapeutic implications may develop from ncRNAs, as agents blocking BCR signaling might be expected to affect the levels of ncRNAs. Monitoring ncRNA molecule levels in patients treated with BCR inhibitors might become a useful surrogate marker for the capacity of the drug to interfere with BCR signaling in the lymphoid compartment. Therefore, regulation by ncRNAs may add a direct therapeutic target in the future.

**Table 1:** ncRNAs in normal B-cell development.

**Type of ncRNA**	ncRNA	Upregulated/Downregulated	Target	Role in B-Cell Development	References
**miRNA**	miR-150	Upregulated	MYB/FOXP1	MiR-150 is mainly expressed in the lymph nodes and spleen and is highly upregulated during the development of mature T and B cells. It regulates FOXP1 in mature B cells. Expression of miR-150 is sharply upregulated in immature B cells. Premature expression of miR-150 blocked the transition from pro-B to pre-B cells. In progenitor B cells, miR-150 targets include the transcription factor MYB. Precise levels of MYB are required for normal haematopoiesis and B-cell development. Loss of MYB leads to a dramatic loss in B cells.	[58,59,60,61]
**miRNA**	miR-17-92 cluster (miR-17, miR-18a, miR-19a, miR-20a, miR-19b-1, miR-92-1)	Upregulated	PTEN/BIM	Overexpression of this cluster results in lympho-proliferative and autoimmune phenotypes due to reduced phosphatase and tensin homolog (PTEN) and Bcl-2-like 11 (Bim) protein expression.	[62,63,64,65,66]
**miRNA**	miR-34a	Upregulated	FOXP1	MiR-34a regulates B-cell differentiation from pro-B cell to pre-B cell. Upregulation of miR-34a at the pro-B cell stage resulted in downregulation of Foxp1 protein, a transcriptional regulator required for B-cell differentiation.	[67,68]
**miRNA**	miR-126	Upregulated	IRS1	MiR-126 has a crucial role in in B-cell lineage commitment. Overexpression of miR-126 in an immature B-cell line and in mouse hematopoietic stem and progenitor cells (HSPCs) induced B-cell differentiation through the regulation of IRS1.	[69]
**miRNA**	miR 212/132 cluster	Upregulated	SOX4	The miR-212/132 cluster is an important regulator of hematopoietic stem cell function, inflammation and proliferation during wound healing. It inhibits the pre-pro-B-cell to pro-B-cell transition by targeting SOX4, which is a crucial mediator of pro-B cell development.	[70,71,72,73,74]
**miRNA**	miR-155/181b	Upregulated	AID	AID expression is required during the B-cell somatic hypermutation process of immunoglobulin genes and its loss leads to the impairment of class switching.	[77,78,79,80,81,82,83,84,85]
**miRNA**	miR-155-5p	Upregulated/ Downregulated	CD10/PU.1	Important to germinal-center (GC) regulation. MiR-155-5p negatively regulates the expression of CD10, a marker of GC cells. Loss of miR-155-5p leads to reduced IgG1 switching by targeting PU.1 transcription factor. This in turn leads to PAX5 downregulation, enabling plasma cell commitment.	[81,82,83]
**miRNA**	miR-125b-5p	Upregulated	PRDM1/IRF4	MiR-125b promotes B lymphocyte diversification in GC by inhibiting premature utilization of essential transcription factors for plasma cell differentiation.	[86]
**miRNA**	miR-9, let-7 and miR-30 families	Downregulated/ Upregulated	PRDM1 BCL6	These miRNA families regulate transcription factors that promote GC B-cell survival and differentiation. They also regulate GC formation and terminal B-cell differentiation.	[87,88,89]
**miRNA**	miR-21	Downregulated	PRDM1 binds to primary miR-21 and represses its translation	The primary miR-21 transcript is a direct target of PRDM1-dependent repression; therefore, it is repressed during interleukin (IL)-21-driven plasma cell differentiation.	[90]
**miRNA**	miR-217	Upregulated	BCL6	A positive regulator of the GC reaction; promotes mature B-cell lymphomagenesis.	[91]
**miRNA**	miR-142-5p	Upregulated	BAFFR	Critical for the development and homeostasis of lymphocytes.	[92]
**miRNA**	miR-148a	Upregulated	BACH2/MITF/PTEN/BIM	An important player in the regulatory network controlling terminal plasma cell differentiation.	[93,94]
**LncRNAs**	LEF1-AS1, SMAD1AS1, MYB-AS, TC-436K13.6	Upregulated/ Downregulated	RAG2/VPREB1/DNTT/LEF1/SMAD1/MYB	Early B-cell development-specific genes, expressed in pro-B, pre-B and immature B cells (e.g., RAG2, VPREB1, DNTT, LEF1, SMAD1, MYB), were associated with LEF1-AS1, SMAD1-AS1 and MYB-AS1 antisense transcripts, as well as with the intergenic transcript CTC-436K13.6.	[158]
**LncRNAs**	OIP5AS, MME-AS1, CRNDE	Upregulated/ Downregulated	KIF23/PLK4/CENPE	Specifically expressed in the proliferative stages of B-cell development, i.e., pro-B, pre-B, centroblasts and centrocytes.	[159,160,161]
**LncRNAs**	PVT1 and multiple uncharacterized lncRNAs: LINC00487, LINC00877, RP11-203B7.2, RP11-132N15.3	Upregulated/ Downregulated	AID/SERPINA/ BCL6	Specifically expressed in GC centroblasts and centrocytes.	[151,152,153]
**lncRNA**	GAS5	Upregulated/ Downregulated	MYC/CDK6/CIAP2/SGK1/MICRORNA-21	GAS5 mediates growth arrest. In proliferating cells, GAS5 is repressed by active mTOR, miR-21 and DNA methylation. In growth arrested cells, GAS5 levels are increased and negatively influence growth- and apoptosis-associated genes, such as MYC, CDK6, cIAP2 and SGK1 (anti-apoptotic), as well as the oncomiR microRNA-21.	[169]

**Table 2:** Aberrantly expressed miRNA in MCL.

**Type of ncRNA**	ncRNA	Expression in MCL	Target	Mechanism/Target/Pathway	References
**miRNA**	miR-17-92 cluster (miR-17, miR-18a, miR-19a, miR-20a, miR-19b-1, miR-92-1)	Upregulated	PTEN/PHLPP2/BIM	A mouse model demonstrated that c-Myc can both activate the expression of the miR-17-92 gene cluster and be regulated by it, suggesting negative regulation between c-Myc and the miR-17-92 gene cluster. Loss of regulation control of this cluster is involved in the development of B-cell non-Hodgkin lymphoma (B-NHL). MiR-17-92 can also inhibit phosphatase and tensin homolog (PTEN), PHLPP2, P21 and Bcl-2-like 11 (Bim) expression, promoting proliferation and suppressing cancer cell apoptosis. High expression levels of the miR-17-92 cluster are also related to poor survival rate in patients with MCL.	[30,96,97,98,99,100,101]
**miRNA**	miR-15a/16-1	Loss/ downregulated	BMI1	MiR-15a/16-1 inhibits cell proliferation, promotes apoptosis of cancer cells and suppresses tumorigenicity by targeting multiple oncogenes associated with short overall survival. MiR-15a/16-1 targets the proto-oncogene Bmi1, which directly regulates pro-apoptotic genes such as Bim and PMAIP1. MiR-15a/16-1 downregulation enhanced the anti-apoptotic potential of MCL.	[102,103,104,105,106,107,108,109,110,111,112]
**miRNA**	miR-34a	Downregulated	MYC/CDK4/6/CCND1/FOXP1/BCL2	MCL patients with downregulated miR-34a had short overall survival associated with poor prognosis. Low expression of miR-34a was associated with high expression of MYC oncogene, which was co-regulated with CDK4/6 and CCND1 to further promote cell-cycle progression and the development of MCL. MiR-34a is involved in the regulation of the tumor suppressor gene TP53 via FOXP1 and BCL2, thus affecting the differentiation and apoptosis of B cells.	[113,114,115,116,117]
**miRNA**	miR-29a/b/c	Downregulated	CDK6/RB1	MiR-29 family members are critical regulators of extracellular matrix (ECM) proteins and signaling pathways associated with fibrosis via targeting of collagens, fibrillins, and elastin. MiR-29 inhibition activated CDK4/CDK6 and RB1 in MCL. Low expression of miR-29 was associated with poor prognosis in MCL.	[104,118,119,120]
**miRNA**	miR-150	Downregulated	MET/FOXP1	MiR-150 inhibits the proliferation and promotes the apoptosis of MCL cells by negatively regulating MET and FOXP1 expression. Premature miR-150 expression severely impairs B-cell development due to a pro- to pre-B-cell transition block, whereas miR-150 deletion promotes B1-cell expansion and increases antibody production. HGF/MET and FOXP1 signaling pathways are considered as treatment targets for B-cell lymphoma, particularly in MCL.	[121,122]
**miRNA**	miR-18b	Upregulated	unknown	MiR-18b decreases the MCL cell line proliferation rate without inducing apoptosis, suggesting that it may render MCL cells resistant to chemotherapy by decelerating the cell proliferation. MiR-18b is associated with poor prognosis.	[123,124]
**miRNA**	miR-20b	Upregulated	unknown	MiR-20b plays a role in survival of patients with MCL. High expression levels of miR-20b are associated with a worse prognosis.	[97,121,125,126]
**miRNA**	miR-223	Downregulated	SOX11	MiR-223 expression is repressed in MCL. It inhibits cell proliferation and promotes G0/G1 accumulation and cell apoptosis. Low expression of miR-223 predicts poorer outcomes in MCL, probably due to its direct targeting of SOX11.	[50,127,128,129,130]
**miRNA**	miR-101	Downregulated	EZH2	Inhibits cell proliferation and induces cell apoptosis of MCL by targeting EZH2. Low miR-101 expression is associated with low overall survival rates.	[131]
**miRNA**	miR-100	Downregulated	mTOR	Inhibits cell proliferation in MCL by targeting mTOR.	[132]
**miRNA**	miR-155-3p	Downregulated	LT-β/non-canonical NF-κB signaling	Overexpression of miR-155-3p led to increased sub-G1 apoptotic cells and reduced cellular viability, demonstrating its tumor suppressive properties.	[134,135,136,137]
**miRNA**	miR-129-2	Downregulated	SOX4	MiR-129-2 has been shown to be a tumor suppressor hypermethylated in epithelial cancers. MiR-129 overexpression inhibited cellular proliferation and enhanced cell death, with concomitant SOX4 mRNA downregulation.	[136]
**miRNA**	miR-26-A-1	Downregulated	EZH2	MiR-26-A-1 is a tumor suppressor. Epigenetic silencing of miR-26-A-1 leads to increased EZH2 levels, which, in turn, translate into a worse outcome.	[138]
**miRNA**	miR-342-3p	Downregulated	Co-regulated with its host gene, EVL	MiR-342-3p is a tumor suppressor co-regulated with its host gene, EVL, by promoter DNA methylation in B-cell lymphomas including MCL. Re-expression of miR-342-3p and EVL and the tumor suppressor function of miR-342-3p were demonstrated by the inhibition of cellular proliferation and increase of cell death.	[139]
**miRNA**	miR-92b/96	Downregulated	PRMT5	Low expression of miR-92b and miR-96 is associated with enhanced PRMT5 translation which is overexpressed in aggressive B-cell NHL, including MCL. Re-expression of miR-92b and miR-96 inhibits PRMT5 translation and alters the growth of MCL cells.	[140,141]
**lncRNA**	MALAT1	Upregulated		MALAT1 is associated with cancer progression, acts through repression of TP53 promoter and increases EZH2 translation. EZH2, in turn, binds a ROR1-AS1 lncRNA, which leads to increased cell proliferation in MCL cell lines.	[164]
**lncRNA**	ROR1-AS1	Upregulated	SOX11/P16	Overexpression of ROR1-AS1 lncRNA promoted growth of MCL cells and resistance to ibrutinib (BTK inhibitor) and dexamethasone treatment through regulation of SOX11 and P16 expression.	[162]
**lncRNA**	FAS-AS1	Upregulated		The promoter region of FAS-AS1 is regulated by EZH2, which is upregulated in MCL. FAS-AS1 modulates alternative splicing of the FAS gene, a central inhibition molecule in the extrinsic apoptosis pathway, thereby downregulating FAS.	[7,165]
**lncRNA**	SNHG4	Upregulated	Co-regulated with eIF4	SNHG4 plays a role in the regulation of gene expression and can bind with eIF4E and regulate protein translation in MCL. Knockdown SNHG4 expression through siRNA inhibits cell proliferation and global protein translation.	[167]
**lncRNA**	SNHG12, FTX, SNHG5, ZNFX1-AS1	Upregulated	Associated with translation machinery via eIF4E/modulation of c-Myc translation	These lncRNAs are associated with translation machinery via eIF4E-RNA-binding motifs in MCL tumor cells. SNHG5 and SNHG12 can also modulate c-Myc translation in MCL cells.	[168]
**lncRNA**	GAS5	Downregulated	eIF4E	Downregulation of the tumor suppressor GAS5, which interacts with translation initiation factor eIF4E to suppress the translation of c-MYC mRNA, resulted in decreased apoptosis levels in MCL cell lines. Overexpression of GAS5 constructs is also sufficient to induce growth arrest in normal and transformed human lymphocytes.	[169]
**lncRNA**	GATA6-AS	Downregulated	GLUT1	GATA6-AS is involved in endothelial–mesenchymal transition. Overexpression of lncRNA GATA6-AS inhibits cancer cell proliferation by downregulating GLUT1 and therefore inhibits glucose uptake in MCL.	[170]
**lncRNA**	LINK-A	Upregulated	Survivin (not directly)	LINK-A lncRNA overexpression promotes cell proliferation, inhibits cell apoptosis and upregulates survivin expression.	[171]
**circRNA**	circCDYL	Upregulated	miR-101	Overexpression of circCDYL in MCL cells promotes malignant proliferation, self-renewal and chemoresistance. Inhibition of circCDYL suppressed cell proliferation in vitro, consistent with its functions in regulating transcription, programmed cell death, cell death and apoptosis. CircCDYL might serve as a sponge for miR-101, targeting EZH2 and further regulating p21 and p27.	[183]
**circRNA**	circ_cgga162	Upregulated	unknown	High circ_cgga162 expression was associated with low overall survival rate.	[184]
**circRNA**	circRAB11FIP1	Downregulated	unknown	Low circRAB11FIP1 expression was significantly associated with *TP53* mutations and with shorter median time to progression.	[180]
